# Incidence Rate and Predictors of Delayed Graft Function Among Adult Kidney Transplant Recipients at a Tertiary Care Hospital in Riyadh, Saudi Arabia

**DOI:** 10.7759/cureus.14985

**Published:** 2021-05-12

**Authors:** Abdulrahman K Almisfer, Salman S Qasim, Meshari A Alqahtani, Mohammad A Alghafees, Almohannad K Alqarni, Najd M AlNowaiser, Abdulrahman Altamimi

**Affiliations:** 1 College of Medicine, King Saud Bin Abdulaziz University for Health Sciences, Riyadh, SAU; 2 Department of Transplantation and Hepatobiliary Surgery, Ministry of the National Guard Health Affairs, Riyadh, SAU; 3 Department of Research Office, King Abdullah International Medical Research Center, Riyadh, SAU

**Keywords:** delayed graft function, transplantation, saudi arabia, donor, kidney

## Abstract

Background

Delayed graft function (DGF) is the most common early postoperative complication of renal transplantation. The occurrence of DGF can lead to both early and late devastating consequences on the allograft’s survival. The risk of developing this complication can increase with certain factors that are related to both the donor and the recipient. In the present study, we aimed to detect the incidence rate of DGF among patients attending a tertiary care hospital in Riyadh, Saudi Arabia, and to investigate potential predictors of DGF.

Materials and methods

This retrospective chart review was conducted at King Abdulaziz Medical City (KAMC), a tertiary care hospital in Riyadh, Saudi Arabia. The inclusion criteria were all patients, 18 years or older, who had renal transplantation from January 1, 2016, to March 31, 2020. Patients who had a second renal transplant, or renal transplantation in a different hospital and were followed up at KAMC were excluded. Patients’ medical records were accessed using the BESTCare electronic system to obtain the patients’ demographic data. A Chi-square test was used to test for the association between a predictor and a delay in graft function.

Results

A total of 344 patients were enrolled in the present study, approximately half of whom were males (56.6%, n=189). Around one-half (49.4%) were aged between 40 and 64 years. The most common cause of renal failure was hypertension, which was found in 117 (35%) patients, followed by diabetes mellitus (DM) in 94 (28.1%) patients. Most organ donors 258 (77.2%) were alive. A total of 23 (6.9%) participants developed DGF. Mycophenolate mofetil (MMF) was found to be significantly associated with DGF (P < 0.001). Those who took MMF (5.9%) had a significantly lower rate of DGF compared to those who did not (36.4%). A significantly higher rate of DGF was seen in patients whose transplants were taken from deceased donors (15.5%) compared to living donor transplants (3.9%). Gender, age, body mass index (BMI), recipient blood type, donor blood type, and cause of renal failure were not associated with DGF.

Conclusions

Only 6.9% of the study’s participants exhibited DGF. The observed rate was lower than the ones detected in the literature. Those who took MMF had a significantly lower rate of DGF compared to those who did not. Transplants of deceased donors (15.5%) showed a significantly higher rate of DGF. Larger multicenter studies are required to further investigate DGF in a region with a high prevalence of organ failure and a higher need for transplantations, such as Saudi Arabia.

## Introduction

Delayed graft function (DGF) is defined as failure of the renal transplant to function immediately, with the need for dialysis in the first post-transplantation week [1]. It is the most common early postoperative complication of renal transplantation [2]. The occurrence of DGF can lead to both early and late devastating consequences on the allograft’s survival, as it has been estimated that DGF decreases renal allograft survival by 40% [2]. The risk of developing this complication can increase with certain factors that are related to both the donor and the recipient. The incidence of DGF has been shown to increase with donor age; young donors have a lower incidence of DGF than donors over the age of 55 [2]. The recipient’s factors that increase the risk of developing DGF include male gender, African American race, diabetes mellitus (DM), longer waiting time on dialysis, and an increased cold ischemia time (CIT) [3]. The diagnosis of DGF can be made through renal transplant biopsy, which is considered a gold standard for the diagnosis of DGF. Moreover, DGF can be identified within the first 24 hours after surgery by low urine output that is not responsive to fluid treatments [2]. In the literature, graft rejection has been linked to DGF [4]. A meta-analysis of 34 studies concluded that patients who developed DGF had an increased risk for graft loss by 41% [5]. Graft's half-life for patients with DGF is shortened regardless of developing acute rejection or not [6]. For standard criteria donor patients who happen to develop DGF, the graft’s half-life is 8.8 years on average, compared to 13 years for patients without DGF [6]. Therefore, it is important to fully understand DGF and its risk factors to minimize graft loss and improve transplant prognosis. In the present study, we aimed to detect the rate of DGF among patients attending a tertiary care hospital in Riyadh, Saudi Arabia. In addition, we aimed to investigate potential predictors of DGF and possible associations between patient-related factors and DGF, such as the demographic characteristics and transplantation history of the patients.

## Materials and methods

This retrospective chart review was conducted at King Abdulaziz Medical City (KAMC), a tertiary care hospital in Riyadh, Saudi Arabia. The inclusion criteria were all patients, 18 years or older, who had renal transplantation from January 1, 2016, to March 31, 2020. Patients who had a second renal transplant, or renal transplantation in a different hospital and were followed up at KAMC were excluded. Patients’ medical records were accessed using the BESTCare electronic system (ezCareTech, South Korea). The main outcome of the study was a delay in graft function. Demographic data including age, gender, body mass index (BMI), and blood type were obtained. The BMI of each patient was categorized into four groups: <18.5 kg/m^2^ as underweight, 18.5-24.9 kg/m^2^ as normal, 25.0-29.9 kg/m^2^ as overweight, ≥30 kg/m^2^ as obese, and ≥35 kg/m^2^ as morbidly obese based on the World Health Organization (WHO) general population classification. The collected transplantation-related data were the cause of renal failure, donor blood type, donor condition, type of immunosuppressant used, and the occurrence of DGF.

The figures were produced by Microsoft Excel 2019 (Microsoft Corporation, WA, USA) and analyzed using the Statistical Package for the Social Sciences (SPSS) version 23.0 (IBM Corporation, NY, USA). For categorical variables, frequency and proportion were used. A Chi-square test was used to test for the association between a predictor and a delay in graft function. A p-value of ≤0.05 was deemed significant. Patients’ confidentiality was ensured by data anonymization using serial numbers instead of medical record numbers. The data were used by the research team members only.

## Results

A total of 344 patients were enrolled in the present study, approximately half of whom were males (56.6%, n=189). In terms of the age at transplantation, 145 (43.4%) were aged between 18 and 39 years, 165 (49.4%) between 40 and 64 years, and 24 (7.2%) 65 years and older. Regarding the BMI, a small proportion of the participants (6.9%, n=23) were underweight, 109 (32.6%) had a normal weight, 91 (27.2%) were overweight, 85 (25.4%) were obese, and 26 (7.8%) were morbidly obese. The most common blood type among recipients was O+ (38.3%, n=128), followed by A+ (24%, n=80), and AB+ (5.1%, n=17). The socio-demographic profile of the participants is displayed in Table 1.

**Table 1:** The socio-demographic profile of the participants BMI: body mass index

Socio-demographic characteristics	n (%)
Gender	
Male	189 (56.6)
Female	145 (43.4)
Total	334 (100)
Age at time of transplantation	
18-39 years	145 (43.40)
40-64 years	165 (49.40)
65 years and older	24 (7.20)
Total	334 (100)
BMI	
Underweight (BMI < 18.5)	23 (6.90)
Normal (18.5 ≥ BMI < 25)	109 (32.60)
Overweight (25 ≥ BMI < 30)	91 (27.20)
Obese (30 ≥ BMI < 35)	85 (25.40)
Morbidly obese (BMI ≥ 35)	26 (7.80)
Total	334 (100)
Recipient blood type	
AB+	17 (5.10)
AB-	3 (0.90)
A+	80 (24.00)
A-	11 (3.30)
B+	75 (22.50)
B-	9 (2.70)
O+	128 (38.30)
O-	8 (2.40)
Missing	3 (0.90)
Total	334 (100)

The most common cause of renal failure was hypertension, which was found in 117 (35%) patients, followed by DM in 94 (28.1%) patients, and glomerular nephrotic syndrome in 26 (7.8%) patients. As for the blood type of donors, the most commonly reported blood types were O+ in 151 (45.2%) patients, followed by B+ in 51 (15.3%) patients, and A+ in 48 (14.4%) patients. Regarding the condition of organ donors, 258 (77.2%) were alive, 72 (21.6%) were deceased, and the condition of 4 (1.2%) organ donors was unknown. As for the post-transplantation immunosuppression therapy, 104 (31.1%) patients took prednisolone, 326 (97.6%) were given tacrolimus (Prograf), and 323 (96.7%) were given mycophenolate mofetil (MMF) (CellCept). A total of 23 (6.9%) participants documented DGF. The complete transplantation history of the patients is illustrated in Table 2.

**Table 2:** Transplantation history of the patients

Outcome variables	n (%)
Cause of renal failure	
Hypertension	117 (35)
Diabetes mellitus	94 (28.1)
Glomerular nephrotic syndrome	26 (7.8)
Glomerular nephritic syndrome	26 (7.8)
Polycystic kidney disease	18 (5.4)
Obstructive uropathy	6 (1.8)
Intra-renal acute kidney injury	6 (1.8)
Pre-renal acute kidney injury	5 (1.5)
Post-renal acute kidney injury	1 (0.3)
Missing	35 (10.5)
Total	334 (100)
Donor blood type	
AB+	3 (0.9)
A+	48 (14.4)
A-	5 (1.5)
B+	51 (15.3)
B-	4 (1.2)
O+	151 (45.2)
O-	15 (4.5)
Missing	57 (17.1)
Total	334 (100)
Organ donor condition	
Deceased donor	72 (21.6)
Living donor	258 (77.2)
Missing	4 (1.2)
Total	334 (100)
Post-transplantation immunosuppression	
Prednisone	104 (31.1)
Tacrolimus (Prograf)	326 (97.6)
Mycophenolate mofetil (CellCept)	323 (96.7)
Imuran (Azathioprine)	1 (0.3)
Delayed graft function	
Yes	23 (6.9)
No	310 (92.8)
Missing	1 (0.3)
Total	334 (100)

As shown in Table 3, MMF (CellCept) was found to be significantly associated with DGF (P <0.001). Those who took MMF (5.9%) had a significantly lower rate of DGF compared to those who did not (36.4%). Organ donor condition was also seen to be significantly linked to DGF (P <0.001). A significantly higher rate of DGF was seen in patients whose transplants were taken from deceased donors (15.5%) compared to living donor transplants (3.9%). Gender, age, BMI, recipient blood type, donor blood type, and cause of renal failure were not associated with DGF.

**Table 3:** The association between participant's socio-demographic characteristics and delayed graft function

Socio-demographic characteristics	Delayed graft function		P-value
	Yes, n (%)	No, n (%)	
Gender			
Male	11 (5.8%)	178 (94.2%)	0.370
Female	12 (8.3%)	132 (91.7%)	
Age at time of transplantation			
18-39 years	12 (8.3%)	132 (91.7%)	0.631
40-64 years	10 (6.1%)	155 (93.9%)	
65 years and older	1 (4.2%)	23 (95.8%)	
BMI			
Underweight (BMI < 18.5)	2 (8.7%)	21 (91.3%)	0.699
Normal (18.5 ≥ BMI < 25)	6 (5.6%)	102 (94.4%)	
Overweight (25 ≥ BMI < 30)	9 (9.9%)	82 (90.1%)	
Obese (30 ≥ BMI < 35)	5 (5.9%)	80 (94.1%)	
Morbidly obese (BMI ≥ 35)	1 (3.8%)	25 (96.2%)	
Blood type			
AB+	1 (5.9%)	16 (94.1%)	0.687
AB-	0 (0%)	3 (100%)	
A+	3 (3.8%)	77 (96.3%)	
A-	1 (9.1%)	10 (90.9%)	
B+	8 (10.8%)	66 (89.2%)	
B-	1 (11.1%)	8 (88.9%)	
O+	7 (5.5%)	121 (94.5%)	
O-	0 (0%)	8 (100%)	
Cause of renal failure			
Hypertension	8 (6.8%)	109 (93.2%)	0.918
Diabetes mellitus	6 (6.4%)	88 (93.6%)	0.770
Glomerular nephrotic syndrome	4 (15.4%)	22 (84.6%)	0.083
Glomerular nephritic syndrome	1 (4%)	24 (96%)	0.537
Polycystic kidney disease	2 (11.1%)	16 (88.9%)	0.486
Obstructive uropathy	0 (0%)	6 (100%)	0.496
Intra-renal acute kidney injury	1 (16.7%)	5 (83.3%)	0.352
Pre-renal acute kidney injury	1 (20%)	4 (80%)	0.253
Post-renal acute kidney injury	0 (0%)	1 (100%)	0.783
Post-transplantation immunosuppression			
Prednisone	4 (3.8%)	100 (96.2%)	0.138
Tacrolimus (Prograf)	22 (6.8%)	303 (93.2%)	0.528
Mycophenolate mofetil (CellCept)	19 (5.9%)	303 (94.1%)	< 0.001
Imuran (Azathioprine)	0 (0%)	1 (100%)	0.785
Other	3 (21.4%)	11 (78.6%)	0.029
Organ donor blood type			
AB+	0 (0%)	3 (100%)	0.886
AB-	2 (4.3%)	45 (95.7%)	
A+	0 (0%)	5 (100%)	
A-	4 (7.8%)	47 (92.2%)	
B+	0 (0%)	4 (100%)	
B-	8 (5.3%)	143 (94.7%)	
O+	8 (5.3%)	143 (94.7%)	
O-	0 (0%)	15 (100%)	
Organ donor condition			
Deceased donor	11 (15.5%)	60 (84.5%)	< 0.001
Living donor	10 (3.9%)	248 (96.1%)	

## Discussion

It is known that aging can lead to a decrease in renal function through several mechanisms [7]. With advancing age, a decline in both nephron size and quantity is seen, in addition to tubulointerstitial changes, glomerular basement membrane thickening, and glomerulosclerosis [8]. These age-related histologic changes are mainly referred to as nephrosclerosis [9]. A study of healthy kidney donors showed nephrosclerotic changes in only 2.7% of the biopsies taken from donors less than 30 years old, 58% from donors 60-69 years old, and 73% from donors over 70 years old [7]. This observed deterioration in renal function can be accelerated by hypertension and DM, leading to chronic kidney disease (CKD), a progressive loss of kidney function, and eventually requiring renal transplantation. Regarding age and its association with DGF, a retrospective cohort study involving 449 recipients concluded that age of over 55 for a recipient was a risk factor for developing DGF [10]. Likewise, another retrospective analysis of 507 kidney transplants showed similar results with the recipient’s age being over 54 [11]. Our findings showed that the recipient’s age at the time of renal transplantation had no association with DGF, which is inconsistent with the literature [11,12]. Similar to age, the recipient’s gender in the present study showed conflicting results. Recent studies reported that male recipients had an increased risk of developing DGF regardless of the donor’s gender [12], while female recipients were less likely to develop DGF due to the impact of gender-specific hormones [13]. These results are inconsistent with the present study, which showed no association between the gender of recipients and DGF.

End-stage renal disease (ESRD) occurs when CKD reaches an advanced state. In ESRD, the kidneys are no longer able to work as they should to meet the body's demands. Certain risk factors such as DM, hypertension, glomerulonephritis, and genetic diseases like adult polycystic kidney disease increase the likelihood of developing ESRD. Globally, the most common risk factor for ESRD is DM [14]. In the present study, the main cause of ESRD was hypertension, which was found in 117 (35%) patients. Similar figures were reported in studies conducted in Indonesia and Turkey where hypertension was reported in 37.4% and 47% of the patients, respectively [15,16]. In comparison, studies conducted in Australia and the United States of America (USA) found that hypertension was the cause of ESRD in only 4.4% and 5.4% of the patients, respectively [17,18]. The second leading cause of ESRD in our patients was DM, which was found in 94 (28.1%) patients. This is similar to figures reported in Indonesia, where ESRD was caused by DM in 26% of the patients [15]. However, studies conducted in other countries reported a significantly lower percentage compared to the present study [16,17,19]. These differences are probably related to early screening, patients’ compliance to medications, and lifestyle modification. Moreover, since hypertension and DM are controllable diseases, focusing on preventive measures, early screening, and compliance to the treatment plan can slow the progression of CKD to ESRD. In the present study, glomerulonephritis was the third most common cause of ESRD, which was seen in 26 (7.8%) patients. This figure is considered very low compared to the values reported in Australia, Nigeria, and the USA, where glomerulonephritis was the cause of ESRD in 47%, 43%, and 29% of the patients, respectively [17-19]. This significant difference could be multifactorial since glomerulonephritis is caused by a variety of conditions ranging from inherited conditions, infections that affect the kidneys, and autoimmune diseases.

Kidney transplant is the most appropriate renal replacement therapy for patients with ESRD in terms of survival and quality of life. Renal transplants are divided into living or deceased donor transplants [17]. A living donor transplant is when a kidney from a living donor is removed and placed into a recipient whose kidneys no longer function properly, while a deceased donor transplant is taken from an individual who has recently died. Regarding the condition of organ donors, we found that 258 (77.2%) of the transplants in the present study were living donor transplants, and 72 (21.6%) were deceased donor transplants. Likewise, a local study conducted in Jeddah, one of the largest cities in the country, reported similar values with 77.3% of kidney transplants being from living donor transplants and 22.7% from deceased donor transplants [20]. In other studies conducted in Turkey and Indonesia, nearly all recipients received kidneys from living donors [16,17], while other studies did not report the involvement of cadaveric transplants in their kidney transplant programs [17-19]. This could be attributed to the superiority of living donor transplants over deceased donor transplants and living donor transplants being more available in some countries compared to others.

Maintenance immunosuppressive therapy is administered to almost all renal transplant recipients to help prevent acute rejection and the loss of the renal allograft. Immunosuppressive agents are available in various combination regimens and include glucocorticoids (primarily oral prednisone), azathioprine, MMF, enteric-coated mycophenolate sodium (EC-MPS), cyclosporine, and tacrolimus [21]. According to the Kidney Disease: Improving Global Outcomes (KDIGO) Clinical Practice Guideline, several randomized controlled trials and meta-analyses demonstrated >90% allograft survival at one year and acute rejection rates of <20% with triple immunosuppressive therapy [22]. The regimen consists of a calcineurin inhibitor (cyclosporine or tacrolimus), an antimetabolite (azathioprine or MMF), and glucocorticoids [21]. In the present study, similar to the KDIGO clinical practice guidelines for kidney transplantation, roughly one-third of our patients were given prednisolone, 326 (97.6%) were given tacrolimus (Prograf), and 323 (96.7%) were given MMF (CellCept).

Regarding DGF, various studies used different definitions for DGF, such as the requirement for dialysis within one week of transplantation, an increase in serum creatinine in the first 24 hours by 43 mmol/L, or a decrease in urine output by 30 mL/hour in the first 24 hours [17]. The most widely accepted definition for DGF is requiring dialysis in the first week post-transplantation. In the present study, we found that 23 (6.9%) of the recipients documented DGF. This value is considered lower than the data in a study conducted in India that involved both deceased and live donor recipients, where 17.2% of the recipients documented DGF [23]. This higher rate of DGF could be due to inadequate treatment of rejection episodes secondary to financial constricts. However, the proportion of patients who exhibited DGF in the present study is slightly higher than the figures reported among Australian live donor recipients, where only 2.3% of the recipients documented DGF [17]. These differences could be attributed to the fact that living donor transplants have superior outcomes compared to deceased donor kidney transplants. The factors that contribute to better function include better organ quality, well-organized surgical conditions, and a reduced ischemia time. Furthermore, the differences seen in the risk factors for developing DGF, such as BMI of the donor, the side of the organ, and the total ischemia time could lead to different outcomes and a reduced rate of DGF [17].

It is known that expanded criteria donors (ECDs), donors over the age of 60, contribute to the risk of DGF. In the present study, most donors were ECDs. Despite that, only 6.9% of the study’s participants exhibited DGF, and among the deceased donor category, only 15.5% of recipients exhibited DGF. A 2019 study of a similar sample size reported a 43.5% incidence rate of DGF among kidney recipients from ECDs who were deceased [24], it also added that prolonged CIT and older donor age were risk factors for DGF. KAMC’s relatively lower DGF rate for deceased donor kidney transplants may be attributed to the standards followed in preserving the kidney and transplanting it as soon as possible; this results in the shortening of CIT as much as possible. Other factors may also be involved, such as selecting donors with fewer comorbidities, younger donors, and donors with normal BMI.

The present study showed a significant difference in the rate of developing DGF among living donor transplants when compared to deceased donor transplants. This result is not unexpected, given the large body of evidence supporting deceased donor transplants being a notable risk factor for DGF [24,25]. In addition to deceased donor transplants, other factors may contribute to DGF. Those factors can be donor-related, such as increasing age and female gender; recipient-related, such as BMI and male gender; measures taken to preserve the graft, such as CIT and warm ischemia time (WIT); and transplant-related factors such as ABO incompatibility and post-transplantation immunosuppression [26]. With respect to gender, the present study found that males and females were equally likely to develop DGF. This result is not supported by the literature [26]. Analysis on DGF recipient data from the United Network for Organ Sharing (UNOS) of more than 100,000 participants spanning 14 years revealed that the male gender of the recipients was highly associated with DGF compared with female recipients, demonstrating the protective effect of the female gender (Abstract: M Levine, A Thomasson, D Aufhauser, R Redfield, P Abt, P Reese. Gender Differences in Kidney Transplant Delayed Graft Function Are Independent of Donor-Recipient Size Mismatch. 2015 American Transplant Congress; May 5, 2015). This discrepancy may be attributed to the study’s smaller sample size and the involvement of a single center in conducting the present study.

There was no significant association in the present study between age and DGF among the study’s three designated age groups. While some studies recognized the effect of donor age on the risk of DGF; by probable way of being more susceptible to the damaging effect of CIT [24,26], there was no conclusive evidence on whether or not recipient age increases the risk of DGF. This may be explained in light of the physiology of the kidney, as it requires perfusion to start producing urine, and recipient age does not influence the vascularity as much as major comorbidities like DM or hypertension do.

The present study did not reveal any significant association between any of the blood groups and DGF. It is notable to mention that all transplants in this study involved ABO compatible donors and recipients; transplant-related DGF by way of ABO incompatibility, therefore, could not be assessed. However, several studies shed light on ABO incompatibility as a risk for DGF rather than recipient blood group per se [26].

It is well established in the literature that an increase in BMI is significantly associated with DGF [26]. A 2011 study on a large data set from the Scientific Registry of Transplant Recipients revealed that a one standard deviation increase in pre-transplant BMI is associated with a 35% higher risk of DGF. Compared to the normal BMI group: overweight, obese, and morbidly obese groups had 30%, 42%, and 118% higher risks of DGF, respectively [27]. The present study, however, showed no significant difference in DGF rates across pre-transplant BMI groups; possibly due to the small sample size and the study being conducted in a single center.

Anti-thymocyte globulin (ATG) induction before transplantation, followed by a triple immunosuppressive regimen (MMF, prednisone, and tacrolimus) post-transplantation is common practice at KAMC. Studies have shown the effectiveness of immunosuppressive therapy, encompassing triple therapy and IL-2 receptor antibody basiliximab in reducing the rates of DGF among kidney recipients, and though the induction used differs from that of KAMC, both ATG and basiliximab are safe and effective options [28]. Triple immunosuppressive therapy and ATG induction were given to all patients in the present study. Therefore, ascertaining their effect on DGF was not possible. Among the deceased donor category, however, only 15.5% of the recipients exhibited DGF, while a 2019 study of a similar sample size reported a 43.5% incidence rate of DGF among recipients of deceased donor kidneys [24]. The immunosuppressive regimen given to patients at KAMC may have a role in this reduced rate. It is worth mentioning that in addition to MMF playing a major role in reducing DGF rates, there is evidence that it slows tubular atrophy progression and interstitial fibrosis [29], which are renal pathologies that significantly contribute to the development of ESRD.

There were some limitations to the present study. First, due to the retrospective nature of the study, associations were evaluated instead of causation in terms of the predictors of DGF. Second, the population size was relatively small compared to global multicenter studies. Although a significant association between MMF and the risk of DGF was found, this association may not be a true reflection of reality, given the limited number of participants in each group of the treatment regimens. Lastly, due to limitations in the BESTCare system, potential pre-operative predictors and baseline parameters such as creatinine levels and CIT were not collected.

## Conclusions

Although most donors were ECDs, only 6.9% of the study’s participants exhibited DGF. The observed rate was lower than the ones detected in the literature. Those who took MMF had a significantly lower rate of DGF compared to those who did not. Organ donor condition was significantly linked to DGF. A significantly higher rate of DGF was seen in patients whose transplants were taken from deceased donors (15.5%) compared to living donor transplants (3.9%). DGF remains a complex dilemma that is influenced by far more than the baseline predictors, which is evident by the conflicting reports in the literature. Larger multicenter studies are required to further investigate DGF in a region with a high prevalence of organ failure and a higher need for transplantations, such as Saudi Arabia.
